# Severe fever with thrombocytopenia syndrome with re-infection in China: a case report

**DOI:** 10.1186/s40249-021-00877-6

**Published:** 2021-06-29

**Authors:** Shou-Ming Lv, Chun Yuan, Lan Zhang, Yu-Na Wang, Zi-Niu Dai, Tong Yang, Ke Dai, Xiao-Ai Zhang, Qing-Bin Lu, Zhen-Dong Yang, Ning Cui, Hao Li, Wei Liu

**Affiliations:** 1 Graduate School of Anhui Medical University, Hefei, 230032 People’s Republic of China; 2 Beijing Institute of Microbiology and Epidemiology, State Key Laboratory of Pathogen and Biosecurity, 20 Dong-Da Street, Fengtai District, Beijing, 100071 People’s Republic of China; 3 The 990 Hospital, People’s Liberation Army, Xinyang, 464000 People’s Republic of China; 4 College of Life Sciences, Fujian Agriculture and Forestry University, Fuzhou, 350002 People’s Republic of China; 5 School of Public Health, Peking University, Beijing, 100191 People’s Republic of China

**Keywords:** Severe fever with thrombocytopenia syndrome, Re-infection, Tick-borne infectious disease

## Abstract

**Background:**

Severe fever with thrombocytopenia syndrome (SFTS), an emerging tickborne infectious disease caused by a novel banyangvirus (SFTS virus, SFTSV), was endemic in several Asian countries with a high mortality up to 30%. Until recently, SFTSV-associated re-infection have not been reported and investigated.

**Case presentation:**

A 42-year-old female patient was identified as a case of SFTS with re-infection, with two episodes of SFTSV infection on June 2018 and May 2020. The diagnosis of SFTS was confirmed by detection of SFTSV RNA in the blood samples using real-time reverse-transcription polymerase chain reaction and antibodies specific for SFTSV using enzyme linked immunosorbent assay. The changes of viremia and antibody response differed between the two episodes. Phylogenetic analysis showed the two viral genome sequences were in the same clade, but showing 0.6% dissimilarity of the nearly whole nucleotide sequence. Analysis of clinical data revealed that the second episode showed milder illness than that of the first episode.

**Conclusions:**

Epidemiological and clinical findings, viral whole genomic sequences, and serological evidence, provided evidence for the re-infection of SFTSV rather than prolonged viral shedding or relapse of the original infection. The patients with re-infection of SFTSV may be at high odds of clinically inapparent or mildly symptomatic. More attention should be directed towards the long-term follow up of the recovered patients in the future, to explicitly acquire the decay profile of their immunity response.

**Graphic abstract:**

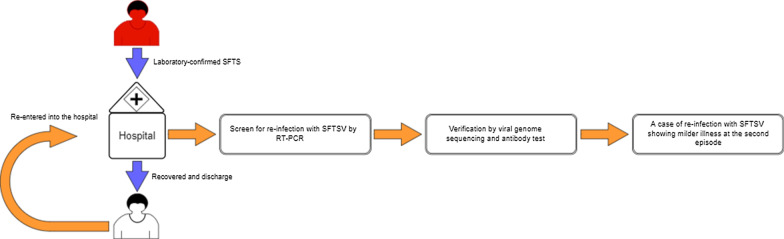

**Supplementary Information:**

The online version contains supplementary material available at 10.1186/s40249-021-00877-6.

## Background

Severe fever with thrombocytopenia syndrome (SFTS) is an emerging infectious disease caused by a novel phlebovirus (SFTS virus, SFTSV) in the family Phenuiviridae of the order Bunyavirales [1]. The disease was identified first in China in 2010 and subsequently in the Republic of Korea and Japan in 2012 [1–3]. So far, the number of SFTS cases has increased and the geographic distribution has expanded consistently, with the cumulative case numbers attaining 11995 in China by 2018, 866 in the Republic of Korea by 2018, and 467 in Japan by August 2019, according to the most recent updates [4]. The mortality rate of SFTS was high, up to 30% at early epidemic period [5]. There has been rare concern as to whether SFTS re-infection occurs, as studies have showed that most of the surviving patients developed neutralizing antibodies after infection of SFTSV [6, 7]. Even antibody titer declined in recovered patients, there is cellular and humoral immune memory that can be stimulated to protect the patient from re-infection [8].

Since the year of first report of SFTS, we have performed a prospective study on SFTS patients in one reference hospital in Xinyang [9], the most severely afflicted region by SFTS in China. All the SFTS patients who were re-entered into the hospital were picked and tested for their re-infection of SFTSV by performing real-time reverse-transcription polymerase chain reaction (RT-PCR) [10]. Here, we describe the first case of re-infection of SFTSV (in a 42-year-old female) in China, and report the clinical and epidemiological findings, as well as characteristics of viral genomic sequences and antibody responses.

## Case presentation

### Disease history and clinical examination

**Fig. 1:**
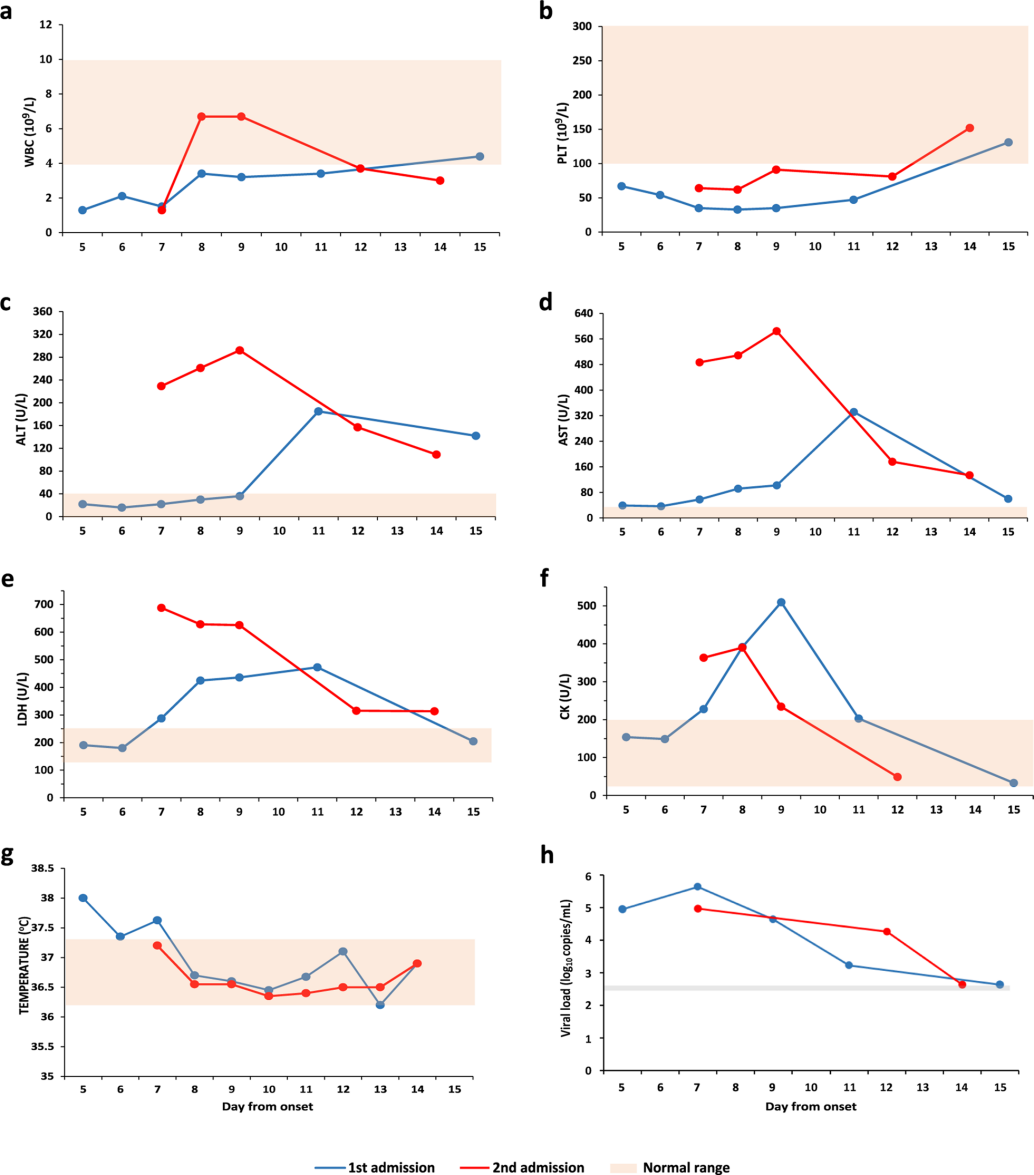
Dynamics of laboratory indicators for two episodes of SFTSV infection from the same patient. **a** White blood cell (WBC) count; **b** Platelet (PLT) count; **c** Alanine aminotransferase (ALT); **d** Aspartate aminotransferase (AST); **e** Lactate dehydrogenase (LDH); **f** Creatine kinase (CK); **g** Body temperature; **h** Viral load were shown. The orange area indicates the normal range; the gray line indicates the detection limit of SFTSV RNA

The patient was a 42-year-old female farmer without underlying conditions residing in Xinyang city, Henan province of China. During the first episode in June 2018, she was admitted into the PLA 990 hospital with acute fever (temperature, up to 39.0 °C), fatigue, nausea, and myalgia for five days. Before this disease, she had taken daily farming work in the field, but reported no history of tick bite during the past month. On admission, blood test determined the presence of leukocytosis [white blood cell (WBC) count, 1.3 × 10^9^/L] and thrombocytopenia [platelet (PLT) count, 67 × 10^9^/L; Table 1]. The diagnosis was confirmed by detection of SFTSV RNA in the blood samples using RT-PCR, and the viral genomic sequence was subsequently obtained. Her conditions during the hospitalization mimicked that of the general pattern from SFTSV infection, i.e., deteriorating shortly after admission, featured by newly developed vomiting (3 times per day), diarrhea (5–6 times per day), persistent decreasing of PLT counts, and increasing of serum alanine aminotransferase (ALT), aspartate aminotransferase (AST), lactate dehydrogenase (LDH), and creatine kinase (CK) levels. The worst situation was observed at 7–10 days after disease onset, and steadily resolve thereafter (Fig. 1). The patient was discharged from hospital at 15 days after disease onset, with all the clinical manifestations resolved and all laboratory abnormalities (except for elevated ALT and AST levels) returning to normal by then. The dynamic evaluation of SFTSV viremia during hospitalization revealed a similar pattern as those of other laboratory indicators, firstly evaluated as 8.8 × 10^4^ copies/ml on admission, increased to peaking level of 4.3 × 10^5^ copies/ml at day 7 after symptom onset, and then reduced to 4.3 × 10^4^ copies/ml, 1.7 × 10^3^ copies/ml at days 9 and 11, respectively (Fig. 1). Viral clearance occurred on day 15 after symptom onset, verified by two consecutive negative RT-PCR tests for SFTSV. The treatment included antiviral therapy with favipiravir and supportive treatment including supplement of electrolytes and dextrose, antipyretics, hepatoprotective, stomach protection, antiemetics, anti-inflammatory, and anticoagulants.

**Table 1:** Laboratory test results of the patient at twice episodes of SFTSV infection

Characteristics	Normal range	First episode		Second episode	
		On admission	Peak or nadir during hospitalization	On admission	Peak or nadir during hospitalization
White blood cell count, × 10^9^/L	4.0–10.0	1.3	1.3	1.3	1.3
Platelet count, × 10^9^/L	100–300	67	33	64	62
Lymphocyte percentage, %	20–40	29.3	12.8	35.6	21.9
Neutrophil percentage, %	50–70	64.6	84.6	58.5	72.7
Hemoglobin, g/L	110–170	120	103	98	96
Alanine aminotransferase, U/L	0–40	22	185	229	292
Aspartate aminotransferase, U/L	0–40	39	332	487	585
Lactate dehydrogenase, U/L	109–245	190	473	688	688
Creatine kinase, U/L	25–200	154	510	363	390
Albumin, g/L	35–55	46.1	34.1	34.7	34.7
Potassium, mmol/L	3.5–5.5	2.63	2.63	3.55	3.55

Her second episode of SFTS developed on May 20, 2020, when the patient was re-admitted into the same hospital due to the symptom of fever (highest temperature 37.8 °C) for 7 days, accompanied by milder clinical manifestations than that of the first episode, such as lower temperature, no vomiting or diarrhea. Again, no tick bite was reported. The dynamic evaluation of laboratory indicators revealed milder thrombocytopenia than the first episode (nadir counts 62 × 10^9^/L vs 33 × 10^9^/L). In contrast, serum levels of ALT, AST, and LDH, were observed with higher peaking levels during the second episode (Table 1). Her temperature returned to normal on day 7 and the physical abnormalities largely resolved on day 14 after symptom onset. The peaking SFTSV viremia appeared lower than that of the first episode, with the viral load estimated as 9.5 × 10^4^ copies/ml at 7 days post disease, decreased to 4.3 × 10^4^ copies/ml on day 12, and to an undetectable level on day 14 after symptom onset (Fig. 1). Similar therapy regimens were administered as the first episode, except for no favipiravir was administered.

### Antibody response

The serum specimens collected at 7, 9, 11, 15 days after symptom onset for the first episode and 7, 12, 14 days after symptom onset for the second episode were tested for IgM and IgG antibodies against SFTSV using enzyme linked immunosorbent assay (ELISA) kit (Wen Ding BioTech Co., Ltd, Nanjing, China). It’s notable that the SFTSV specific IgG antibody remained negative during the hospitalization for the first episode, while was produced at 14 days post disease for the second episode to titer of 1:80. In parallel, IgM turned positive at 9 days post disease for the first episode, while tested to be positive at 7 days with titer of 1:80 for the second episode (Additional file 1: Table S1).

### Virus molecular characterization

**Fig. 2:**
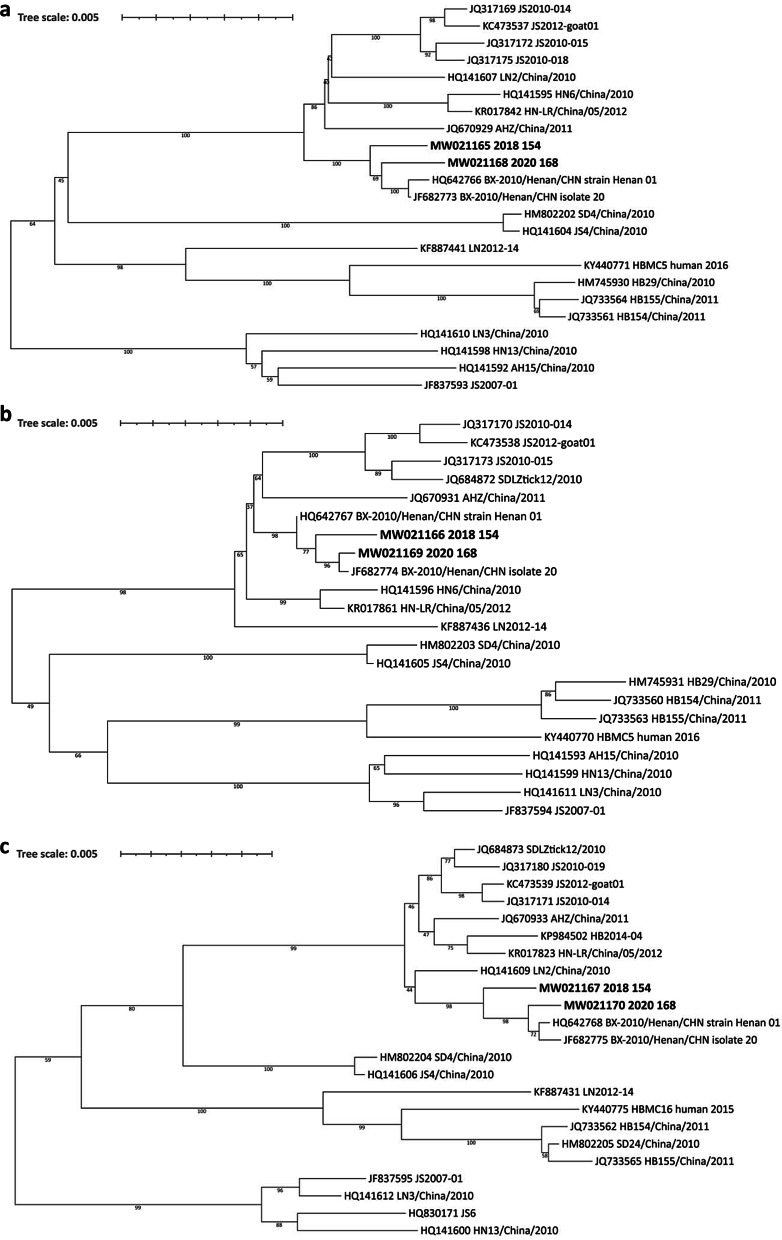
Phylogenetic analysis based on nucleotide sequences of SFTSV. **a** Nearly full-length of the L segments (6258 bp); **b** M segments (3335 bp); **c** S segments (1689 bp) of SFTSV were used for construction of phylogenetic trees. The Maximum Likehood method with the best substitution model (Tamura 3-parameter + G) was conducted using MEGA version 5.0 (http://www.megasoftware.net). Bootstrap analysis of 1000 replicates was applied to assess the reliability of the reconstructed phylogenies. Scale bars indicate estimated evolutionary distance. The strain name of SFTSV was specified. The bold indicates the two SFTSV strains in the study

Whole genome sequencing was performed on two samples that were collected before antiviral therapy from two episodes respectively. Phylogenetic analysis based on nucleotide sequences of L (GenBank accession numbers: MW021165 and MW021168), M (MW021166 and MW021169), or S (MW021167 and MW021170) segments [11], showed that the two viral genome sequences were in the same clade, most closely related to strains identified in Xinyang (Fig. 2). The two virus genomes differ by 72 nucleotides, with 38, 21, and 13 in the L, M, S segments, respectively. Nine different amino acids were identified between the two genomes, including 4 in the RNA-dependent RNA polymerase (RdRp), 2 in glycoprotein precursor (GP), 1 in nucleocapsid protein (NP), and 2 in nonstructural protein (NS).

## Discussion and conclusions

Previous reports have raised questions around the possibility of re-infection with highly lethal viruses such Ebola virus and Lassa virus [12, 13], however, fewer confirmed cases have been reported. In the current study, we reported a case of re-infection with SFTSV, which was supported by epidemiological and clinical findings, as well as whole genomic sequences and serological evidence, rather than prolonged viral shedding or relapse of the original infection. First, whole genome analysis revealed 72 nucleotide differences between the SFSTV strains determined from two episodes. Both were phylogenetically close to the locally circulating strains, fully reflective of the infectious source of this patient. Second, the patient was discharged from hospital only after two consecutive negative detection for SFTSV. Although no examination was performed during the interval between two episodes, persistent existence of SFTSV over two years is impossible. Third, the patient had relatively high viral load with gradual decline for both episodes, and seroconversion of SFTSV IgG during the first episode was lower than the average level which had been derived from other patients treated in the same hospital [14], highly likely related to the second episode of re-infection.

The mutation of the second strains was unlikely to evade the protection elicited from the first infection. Therefore, the comprised immunity to SFTSV that was acquired from the first infection might render the patients succumb to the re-infection, however, no samples were collected during the interval between two episodes, thus no measurement of antibody was available to determine to what extent the antibody had been waned or whether there was immune memory to be stimulated upon re-exposure to the virus.

Based on this finding, we suggest that although occurring with rare occasion, host immunity acquired following primary SFTSV infection might fail to protect patients from re-infection after subsequent exposure to the virus. The second visit of this patient showed milder clinical manifestations and lower viremia, yet higher serum levels of ALT, AST, and LDH, in comparison with the first episode, which phenomenon is beyond expectation. However, the AST/ALT ratio that has been considered as a better indicator of the type of liver injury than changes in AST or ALT alone [15], was higher in the first episode (Additional file 1: Fig. S1). No antibody dependent enhancement (ADE) had ever been reported for SFTSV or any other members in the same family, and we assume this case presented here represent only the untypical clinically apparent infections which presented to health care facilities. In case of re-infection, the patients are at high odds of clinically inapparent or mildly symptomatic. Thus, more attention should be directed towards the long-term follow up of the recovered patients in the future, to explicitly acquire the decay profile of their immunity response.

The current finding has implication that in case of vaccine development, vaccines may not be able to provide lifelong protection against SFTSV, as infection might occur despite a static level of specific antibodies. Still more isolation and cross protection assay is warranted to determine whether these amino acid differences in the G protein defined between two episodes is responsible for the re-infection.

## Supplementary Information

**Additional file 1: Table S1**. The titers of SFTSV specific IgM and IgG antibodies for two episodes of SFTSV infection from the same patient. **Fig. S1**. Dynamic ratio of aspartate aminotransferase and alanine aminotransferases for two episodes of SFTSV infection from the same patient.

## Data Availability

Not applicable.

## Abbreviations

SFTS: Severe fever with thrombocytopenia syndrome
SFTSV: Severe fever with thrombocytopenia syndrome virus
RT-PCR: Real-time reverse-transcription polymerase chain reaction
ELISA: Enzyme linked immunosorbent assay
WBC: White blood cell
ALT: Alanine aminotransferase
AST: Aspartate aminotransferase
LDH: Lactate dehydrogenase
CK: Creatine kinase
RdRp: RNA-dependent RNA polymerase
GP: Glycoprotein precursor
NP: Nucleocapsid protein
NS: Nonstructural protein
ADE: Antibody dependent enhancement
