# Supplementary material for: Severe fever with thrombocytopenia syndrome with re-infection in China: a case report
Source: Infect Dis Poverty. 2021 Jun 29;10:90. doi: 10.1186/s40249-021-00877-6 (PMC8238631; doi:10.1186/s40249-021-00877-6)

**Additional file Data**

**Additional file Table S1 The titers of SFTSV specific IgM and IgG antibodies for two episodes of SFTSV infection from the same patient.**

| Admission | Day from symptom onset | Antibody titer | |
| --- | --- | --- | --- |
|  |  | IgG | IgM |
| 1st | 7 | Negative | Negative |
|  | 9 | Negative | 160 |
|  | 11 | Negative | 320 |
|  | 15 | Negative | 320 |
| 2nd | 7 | Negative | 80 |
|  | 12 | Negative | 320 |
|  | 14 | 80 | 320 |
|  | | | |

**Additional file Fig. S1** Dynamic ratio of aspartate aminotransferase and alanine aminotransferases for two episodes of SFTSV infection from the same patient.

AST, aspartate aminotransferase; ALT, alanine aminotransferase.


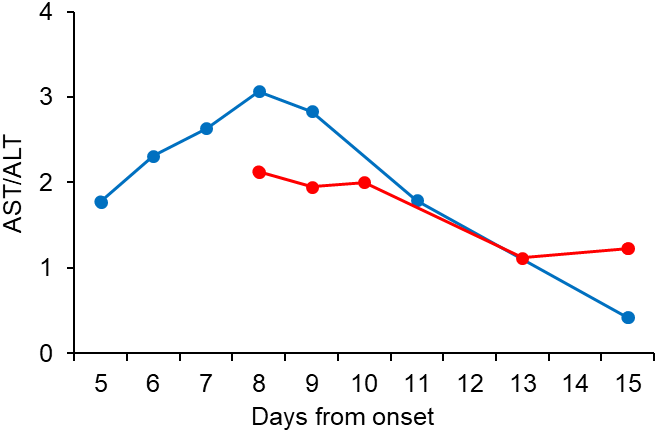

Supplement: Supplementary file 1 — Additional file 1: Table S1. The titers of SFTSV specific IgM and IgG antibodies for two episodes of SFTSV infection from the same patient. Fig. S1. Dynamic ratio of aspartate aminotransferase and alanine aminotransferases for two episodes of SFTSV infection from the same patient. [file 40249_2021_877_MOESM1_ESM.doc]
